# An Enhanced GC/MS Procedure for the Identification of Proteins in Paint Microsamples

**DOI:** 10.1155/2018/6032084

**Published:** 2018-04-01

**Authors:** D. Fico, E. Margapoti, A. Pennetta, G. E. De Benedetto

**Affiliations:** ^1^Laboratorio di Spettrometria di Massa Analitica ed Isotopica, Dipartimento di Beni Culturali, Università del Salento, 73100 Lecce, Italy; ^2^IBAM-CNR, Sede di Lecce, Via per Monteroni, 73100 Lecce, Italy

## Abstract

The chemical characterization of materials used in works of art is extremely useful for gaining a better knowledge of the artistic heritage and to guarantee its preservation. A derivatization GC/MS procedure for the identification of proteins in a microsample from painted works of art has been optimized. The amino acid fraction is derivatized using anhydrous dimethylformamide (DMF) as solvent instead of pyridine (Py), commonly used to facilitate the reaction. Although pyridine is often considered a silylation catalyst, there are many instances in which silylation reactions actually are slower in pyridine than other solvents. In addition, pyridine also may have other undesirable effects such as the promotion of secondary products and other chromatographic anomalies. Using DMF, the formation of artifacts is limited and the derivatization yield of hydrophilic amino acids such as proline and hydroxyproline has improved, thus making the identification of organic paint media more straightforward. The method has been validated and successfully applied to identify the binder of the sample taken from the pictorial cycle of the 12th century monastery of Santa Maria delle Cerrate (Lecce, Italy), thus highlighting the use of eggs as a binding medium.

## 1. Introduction

Proteinaceous paint media, especially those based on egg and milk, have been used over the centuries by painters, due to excellent optical features and stability of the paint films obtained [1, 2]. The chemical characterization of materials used is extremely useful for surveying historical treatments and for gaining a better knowledge of the artistic heritage. This study entails investigating the original constituent materials and those become an integral part of the work of art itself as an effect of aging and restorations. Assessing the state of conservation of a painting is fundamental when choosing a conservative strategy based on both prevention and intervention. Developing analytical techniques for understanding the chemical composition of painting materials and for studying the degradation process is thus of great importance.

In the conservation of paintings, a special care and attention to organic materials is necessary, due to their relatively increased tendency to undergo degradation, transformation, and oxidation process [2]. In fact, the paint layers may exhibit cracking, darkening, yellowing, and loss of stability and cohesion. Milk or casein, animal glue, egg, drying oils (walnut, linseed, and poppy seed oils), plant resins (e.g., sandarac, mastic, and colophony), animal resins, and waxes are the most common organic materials historically used in the Mediterranean area and in Europe. The chemical composition and physical properties of these organic materials (mixtures of organic species consisting of proteins, triglycerides, terpene compounds, sterols, esters, alcohols, hydrocarbons, free acid, etc.) are considerably influenced by environment and aging [1, 2]. A wide range of natural and synthetic organic materials was used for consolidation and restoration [1]. Organic materials were used as binders to disperse pigments and to apply them on all the supports, thereby generating an adherent and elastic film. Proteinaceous materials were thus employed in the tempera technique, drying oil in the oil technique, and beeswax in encaustic painting [1, 2]. The precise study of organic materials in cultural heritage works was investigated on painting cross sections since the early 1970s using staining methods with organic dyes such as naphthol blue black [2] for the specific detection of proteins, allowing an in situ microcharacterization of egg-based or collagen-based materials. After that, spectroscopic techniques were employed for characterization of proteinaceous binders, as well as calorimetric analysis (DSC and TGA) for investigated proteinaceous materials and interference with inorganic materials [3]. Methods based on fluorescence detection HPLC-FD and HPLC-DAD UV diode array detection were employed to study amino acid composition of binders [4], but GC/MS is above all the most common method used for the characterization of organic species in pictorial samples and their behavior with aging.

Different GC/MS analytical procedures for the characterization of proteins [2, 5–8], lipids [9–12], plant resins [13–17], animal resins [18], and waxes [19–21] are described in the literature.

Generally proteins, following ammonia extraction and acid-catalysed hydrolysis, are identified by quantifying the free amino acids by GC/MS after derivatization [22–24]. In this paper, the optimization of derivatization GC/MS procedure for the identification of proteins in the paint microsample is presented. Silyl derivatives are probably the most widely used derivatives for GC applications [25]. However, silylation involves reaction with active hydrogen atoms; for these reasons, all solvents containing or capable of activating these groups (e.g., alcohols, acids, primary and secondary amines, mercaptans, and primary and enolizable ketones) should be avoided. Often, an excess of the silylation reagent itself can act as the solvent, eliminating the need for additional components in the analytical scheme. Hexane, ether, benzene, or toluene is an excellent solvent for the reaction products, but they do not accelerate the rate of reaction. Among polar solvents, pyridine (Py) has been generally used in the identification of organic binders in paints [22, 26, 27]. However, there are many examples in which silylation reactions are actually slow in pyridine, for instance, with hydrophilic amino acids such as proline and hydroxyproline [28]. In addition, pyridine also may have other undesirable effects such as the promotion of secondary products and other chromatographic anomalies [29].

In the present paper, the amino acid fraction obtained after extraction and hydrolysis of different proteinaceous binding media [22] has been derivatized using anhydrous dimethylformamide (DMF) as solvent instead of pyridine. DMF has been used especially for large molecules [26, 28]; in the present case, it is shown to be effective for small molecules such as the amino acids as well. Moreover, by using DMF, we can limit the formation of artifacts [29, 30] and improve the derivatization yield for hydrophilic amino acids such as proline and hydroxyproline.

Reference samples produced by a local restorer were employed to test and validate the analytical procedure. The percentage contents of amino acids, obtained by the proposed procedures, were submitted to correlation analysis with those obtained from egg protein, animal glue, and milk hydrolysis in order to achieve the identification of the proteinaceous binders present in the sample. Finally, the results relevant to the sample from the wall paintings from Santa Maria delle Cerrate (Lecce, Apulia, Italy), a monastery of the first half of the 12th century, are presented.

## 2. Experimental

### 2.1. Reagents

All the solvents were HPLC grade. Hexadecane (I.S.1), triethylamine and *N*-tert-butyldimethylsilyl-*N*-methyltrifluoroacetamide (MTBSTFA) were purchased from Fluka (USA), Merk (Germany), and Sigma-Aldrich (Italy), respectively. All reagents and chemicals were used without any further purification. Ultrapure water (Integra UV, Diessechem, Italy) was used all through. Standard solutions of amino acids purchased from Sigma-Aldrich and containing 2.5 *µ*mol/mL in 0.1 M HCl of each amino acid, alanine (Ala), glycine (Gly), valine (Val), leucine (Leu), isolucine (Ile), proline (Pro), hydroxyproline (Hyp), serine (Ser), threonine (Thr), tyrosine (Tyr), methionine (Met), cysteine (Cys), arginine (Arg), phenylalanine (Phe), histidine (His), aspartic acid (Asp), and glutamic acid (Glu), except for cystine (CySS) whose concentration was 1.25 *µ*mol/mL. Norleucine (Nor) was used as an internal standard (I.S.2). Animal glue was supplied by Kremer Pigmente GmbH & Co. KG (Germany), while egg yolk and milk have been procured by the authors.

### 2.2. Apparatus

Microwave oven model Ethos (Milestone) and GC system gas chromatograph coupled with a 5973 inert single quadrupole mass spectrometer (Agilent Technologies) and equipped with a split/splitless injector were used. The MS transfer line temperature was 280°C, the MS ion source temperature was kept at 230°C, and the MS quadrupole temperature was at 150°C. The mass spectrometer operated in the EI positive mode (70 eV). For the gas chromatographic separation, an HP-5MS fused silica capillary column (5% diphenyl/95% dimethyl-polysiloxane, 30 m, 0.25 mm i.d., 0.25 *µ*m film thickness, J&W Scientific, Agilent Technologies, Palo Alto, CA) with a deactivated silica precolumn (3 m, 0.25 mm i.d., Supelco) was used. The carrier gas (He, purity 99.9995%) was used in the constant flow mode at 1.2 mL/min. For the amino acid analysis, the injector was used in a splitless mode at 250°C. The chromatographic oven was programmed as follows: initial temperature 100°C, isothermal for 2 min, then 4°C/min up to 280°C, and isothermal for 15 min. MS spectra were recorded in a TIC (total ion current) mode.

### 2.3. Mass Spectra Assignment

Mass spectral assignment was based on the direct match with the spectra of both NIST 2002 library and the laboratory library comprising about 200 mass spectra of pure compounds used in art materials.

### 2.4. Samples

#### 2.4.1. Reference Materials

The method was tested also on reference materials for the identification of proteinaceous binders on the basis of the quantitative determination of the amino acid profile. Whole egg (We), animal glue (Ag), and whole milk (Wm) were applied on untreated wood specimens (Table 1); whole egg was slightly beaten to obtain a homogeneous mixture, animal glue was dissolved in warm water, and whole milk was used without any treatment. All solutions were applied alone with a brush on test specimens.

The characterization of proteinaceous material can be affected by inorganic compounds derived from the preparatory or from the pictorial layers. For this reason, interference due to inorganic material was evaluated as follows: approximately 1 part of pigment powder (azurite Cu_3_(CO_3_)_2_(OH)_2_, minium Pb_3_O_4_, and Prussian blue Fe_4_[Fe(CN)_6_]_3_) was added to 2 parts of fluid binder (whole egg, animal glue, and milk) and homogenated (Table 1). For a better comprehension of the pigment-binding interaction mechanisms that occur in general in the real samples, Fe and Ca interference has been studied also using a specimen prepared by mixing red ochre with the different binders (whole egg, animal glue, and whole milk), which were applied on wood specimens primed by a layer of gypsum and animal glue: gypsum and animal glue were mixed in 1 : 1 ratio, and red ochre and binder were in 1 : 2 ratio.

#### 2.4.2. Santa Maria delle Cerrate

The method was also applied on a sample of the wall painting of the Church of Santa Maria delle Cerrate (Puglia, Italy). The decoration of the Church, according to written sources, was built in the second half of the 12th century, and it exhibits stylistic aspects related to Greek-byzantine tradition. The pictorial apparatus develops on central apse and lateral aisles. In particular, the sample was collected from the Saint depicted on the wall of the central apse of the Church, and its weight was 1.8 mg. A multistep chemical sample pretreatment based on the ammonia extraction was required to obtain the proteinaceous fraction (together with the polysaccharide fraction) and to separate it from the lipid-resinous fraction [31].

### 2.5. Analytical Procedure

The analytical procedure described in [22] has been used to separate protein, lipid, and carbohydrates from the same microsample by GC/MS. Quantification of lipid and carbohydrate was carried out as described in the same paper, whereas protein extracts were treated as follows:

- The ammonia extract containing proteins is evaporated to dryness under a stream of nitrogen and then subjected to acidic hydrolysis assisted by microwave (power 500 W) in the vapor phase with 50 *µ*l of 6 N HCl at 160°C for 40 min.
- Bidistilled water (300 *µ*L) and then 5 *µ*L of norleucine (Nor) are added to the acidic hydrolysate.
- The hydrolysate is evaporated to dryness under a stream of nitrogen (repeated twice by adding a few drops of acetone) and amino acid silylated with 20 *µ*L of *N*-tert-butyldimethylsilyl-*N*-methyltrifluoroacetamide (MTBSTFA), 73 *µ*L of DMF (solvent), 2 *µ*L of triethylamine (catalyst), and 5 *µ*L of hexadecane solution at 60°C for 30 min.
- 2 *µ*L of the solution of derivatized amino acids are analyzed by GC/MS.

**Figure 1:**
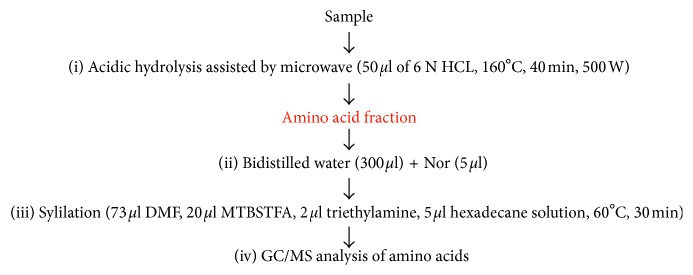
Analytical procedure for quantitative determination of the amino acid profile.

These steps are outlined in Figure 1.

### 2.6. Method Validation

The amino acid stock solution was diluted to obtain a calibration curve ranging from 5 nmol/*µ*L to 30 nmol/*µ*L. Six calibration levels were used, each of which was injected three times. Nor was added to each diluted standard mixture (100 nmol/*µ*L final concentration) as IS. The calibration curve was constructed by plotting the ratio analyte peak area/IS peak area versus analyte/IS concentration ratio for each individual analyte. Lack-of-fit test was used to prove the linear range. The instrumental limit of detection (LOD) and limit of quantification (LOQ) of each amino acid was determined as the minimum amount of analyte producing a chromatographic peak with a signal-to-noise ratio equal to 3 and 10, respectively. Six replicate measurements of reagent blanks spiked with low concentrations of analytes (1 nmol/*µ*L) were performed to obtain the *S*/*N*.

The repeatability and the recovery of the method have been calculated on reference materials. In particular, reference materials were analyzed ten times on the same day and the repeatability was expressed in terms of relative standard deviation (RSD, %), while recoveries were calculated using the following formula: % recovery = 100 × (amount of amino acid determined using DMF solvent)/(amount of amino acid determined using Py solvent). The interference was calculated as recovery (%) = 100 × (amount of amino acids detected in the slurry of the binder and the inorganic material)/(amount of amino acids detected in the binder).

## 3. Results and Discussion

**Figure 2:**
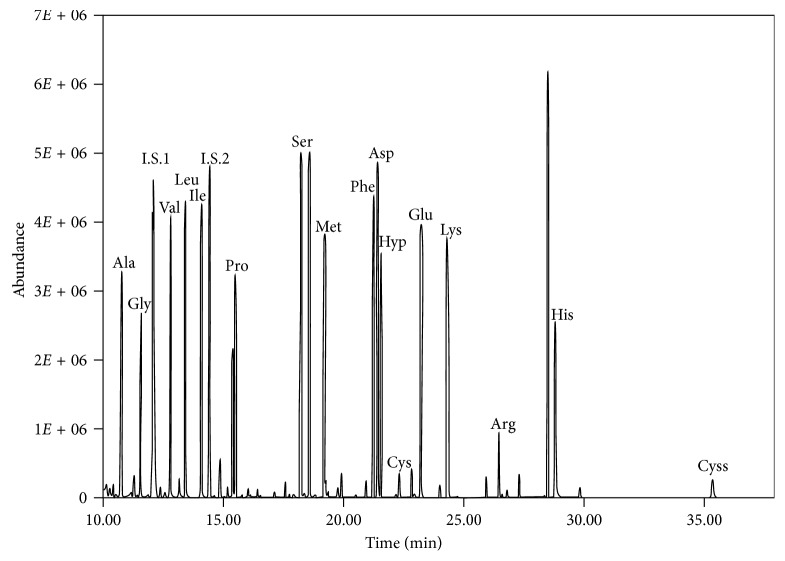
The GC-MS detection of derivatized amino acids with DMF as solvent in TIC mode.

To evaluate the linearity of the GC/MS method, variable volumes of an amino acid standard mixture were separately evaporated and derivatized, using pyridine and DMF as solvents, and the dilution series of amino acids derivatives were subsequently analyzed. The calibration plots showed good linearity for all compounds tested with DMF and pyridine (Table 1). The use of pyridine as solvent gave a linear response with correlation coefficient (*r*^2^) values ranging from 0.71 to 0.99. Also, the technique based on the use of DMF as solvent gave linear response in the range 5 nmol/*µ*L to 30 nmol/*µ*L with *r*^2^ values ranging from 0.80 to 0.99 and instrumental limits of detection ranging from 0.03 to 0.37 ng for the different amino acids. However, comparing the values of the correlation coefficient (*r*^2^) obtained with the two different methods of derivatization, it is observed that for some amino acids, such as proline and hydroxyproline, the *r*^2^ is greater when we use the DMF. In fact, *r*^2^ of the proline is 0.89 with pyridine and 0.99 with DMF, while *r*^2^ of the hydroxyproline is 0.72 with pyridine and 0.95 with DMF (Table 2). In Figure 2 is shown a typical chromatogram, where the peaks of all amino acids derivatized with DMF as solvent are resolved and easily identifiable.

The repeatability was <20.0% for each of the compounds and recoveries were between 81% and 105%. Another important advantage of the derivatization with DMF as solvent is that it is not mandatory to employ methods of sample cleanup on real pictorial samples containing protein binders. High concentrations of inorganic salts present in the pictorial samples can produce chromatographic interference that inhibits the correct identification of the proteins [3, 32, 33]. Recent studies have shown the effectiveness of desalting and concentration techniques based on the pipette tip cleanup [34, 35]. The cleanup step is based on the fact that proteins are bound to the stationary phase of the pipette tip, whereas inorganic salts are unretained. The derivatization with DMF as solvent allows to obtain a significant increase of the signal-to-noise ratio without the use of cleanup treatments of proteins reducing the time for preparation and analysis of the samples. As evidenced by reference material analysis, the presence of pigments, present as salts or oxides (azurite, minium, Prussian blue, red ochre, and gypsum), and added to whole egg, animal glue, and whole milk, did not affect recoveries of amino acids that remained in the range of 74–109% nor the relevant profiles. Amino acid content of the analyzed mixtures, expressed as relative percentages, is summarized in Table 3.

**Figure 3:**
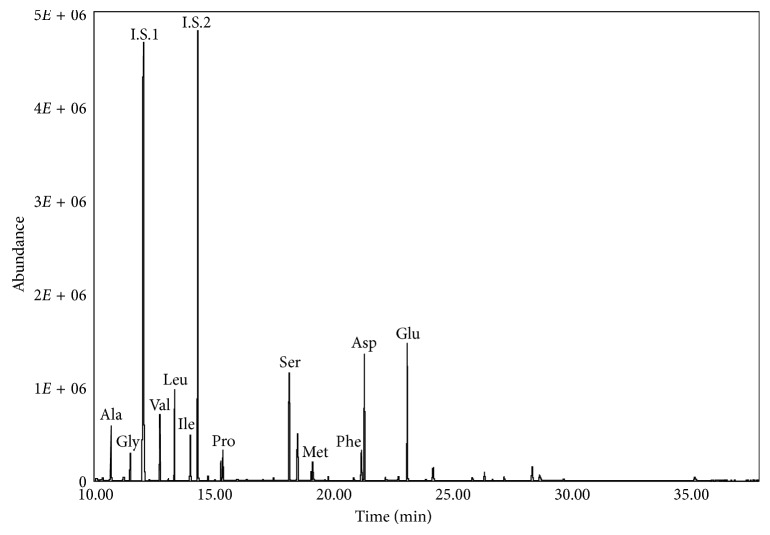
The GC-MS detection of Santa Maria delle Cerrate sample derivatized with DMF as solvent in TIC mode.

The procedure was also effective on a real sample collected from the wall paintings of the Church of Santa Maria delle Cerrate (Italy) and derivatized with the proposed method (Figure 3) [36]. The results of the analysis are presented in Table 4 where the amino acid composition (w/w%) of proteins extracted from the wall painting sample is reported along with literature data of proteinaceous binding media such as egg, animal glue, and milk [37]. The correlation coefficients among the unknown sample and reference proteinaceous materials (*r*^2^) strongly suggest the use of eggs as a binding medium in the sample as *r*^2^ was equal to 0.95. This is confirmed also by the presence of cholesterol in trace, the ratio azelaic acid/palmitic acid (A/P) < 0.1 [2, 5], and by the Σ*D*% < 1.5 [5, 32].

## 4. Conclusion

Proteinaceous paint media especially those based on egg and milk have been used over the centuries by painters due to excellent optical features and stability of the paint films obtained. Chemical characterization of these materials present in the pictorial samples provides a better knowledge of the artistic heritage and allows us to act during restoration and conservation correctly. The GC/MS analytical techniques are best suited for identifying the binding protein used in cultural heritage, and it is based on hydrolysis and derivatization processes. In this paper, the amino acid fraction, obtained following the procedure described above and reported in the literature [22], is derivatized using anhydrous dimethylformamide instead of pyridine as solvent. The enhanced GC/MS procedure for the identification of proteins was validated, and the method demonstrated was precise and accurate. The linearity was tested in the same range for both methods and *R*^2^ coefficients were confrontable while LODs and LOQs calculated for DMF solvent were better than values obtained with Py solvent. Overall, the results show that using DMF as solvent, we can limit the formation of analytical artifacts, get clean chromatograms, and improve the LOQ of hydrophilic amino acids such as proline and hydroxyproline. Sample cleanup treatments could be avoided, thus reducing the analysis time: interference of inorganic salts appears not so severe as to prevent recognition of proteinaceous binders. The protocol was successfully tested on reference materials and on a sample collected from the wall paintings of the Church of Santa Maria delle Cerrate (12th century, Italy).

## Figures and Tables

**Table 1:** List of binders and pigments used as reference materials.

Pigment	Binder			Sample name
	Whole egg	Animal glue	Whole milk	
None	X			We
None		X		Ag
None			X	Wm
Azurite	X			Az-We
Azurite		X		Az-Ag
Azurite			X	Az-Wm
Minium	X			Mi-We
Minium		X		Mi-Ag
Minium			X	Mi-Wm
Prussian blue	X			Pb-We
Prussian blue		X		Pb-Ag
Prussian blue			X	Pb-Wm
Red ochre + gypsum	X	X		Ro/Gy-We/Ag
Red ochre + gypsum		X		Ro/Gy-Ag
Red ochre + gypsum		X	X	Ro/Gy-Wm/Ag

**Table 2:** Linear correlation coefficients (*r*^2^).

Amino acid	Quantitation ion (*m*/*z*)^*∗*^	Retention time (min)			Py					DMF		
			*a*	*b*	Correlation coefficient (*r*^2^)^*∗∗*^	LOD (ng)	LOQ (ng)	*a*	*b*	Correlation coefficient (*r*^2^)^*∗∗*^	LOD (ng)	LOQ (ng)
Ala	158 (100)	10.81	0.003	0.484	0.9666	0.126	0.421	0.003	0.508	0.9973	0.028	0.094
Gly	218 (76)	11.59	0.001	0.376	0.9836	0.115	0.382	−0.041	0.355	0.9882	0.056	0.188
Val	186 (100)	12.78	−0.013	0.613	0.9672	0.190	0.632	0.003	0.616	0.9977	0.030	0.102
Leu	200 (100)	13.42	−0.004	0.677	0.9432	0.201	0.670	−0.007	0.665	0.9986	0.031	0.103
Ile	200 (100)	14.10	−0.006	0.616	0.9631	0.198	0.661	0.003	0.617	0.9981	0.034	0.114
Pro	184 (100)	15.49	−0.009	0.647	0.8909	0.174	0.580	−0.03	0.795	0.9963	0.046	0.154
Ser	288 (100)	18.23	−0.001	0.273	0.9251	0.163	0.543	−0.002	0.43	0.9923	0.056	0.187
Met	292 (83)	19.21	0.003	0.225	0.9595	0.234	0.780	−0.011	0.221	0.9957	0.061	0.203
Phe	302 (100)	21.24	−0.004	0.324	0.9528	0.255	0.850	−0.004	0.331	0.9969	0.059	0.195
Asp	302 (100)	21.42	0.002	0.276	0.9656	0.272	0.905	0.002	0.333	0.8017	0.241	0.804
Hyp	314 (100)	21.55	0.001	0.107	0.7197	0.411	1.369	0.007	0.588	0.9555	0.168	0.561
Glu	432 (100)	23.23	−0.015	0.319	0.9873	0.457	1.522	0.004	0.334	0.8224	0.372	1.241

*Note.* Linear correlation coefficients (*r*^2^), LOD and LOQ of amino acids were derivatized using Py or DMF as solvent. ^*∗*^Number in parentheses indicate % relative intensity of ions; ^*∗∗*^correlation coefficients obtained from linear regression analysis of calibration curve.

**Table 3:** Relative percentage content of amino acids in reference samples and relevant correlation coefficient.

Sample	Amino acids											Correlation		
	Ala	Gly	Val	Leu	Ile	Pro	Met	Ser	Asp	Hyp	Glu	*r* ^2^_Sa-We_	*r* ^2^_Sa-Ag_	*r* ^2^_Sa-Wm_
We	10.3	7.8	9.0	11.6	6.4	6.4	2.6	10.0	16.4	0.0	19.6	1.00	—	—
Ag	13.8	26.0	4.2	5.5	3.0	12.2	1.1	3.5	5.6	14.9	10.2	—	1.00	—
Wm	4.4	3.8	9.6	11.5	7.7	19.2	2.5	3.0	8.7	0.0	29.6	—	—	1.00
Az-We	8.9	7.3	8.8	12.1	6.0	6.9	2.4	10.7	17.0	0.0	20.0	0.99	−0.12	0.67
Az-Ag	14.8	28.0	3.9	5.6	3.2	12.8	1.1	3.6	5.4	11.5	10.0	−0.03	0.99	−0.01
Az-Wm	4.7	3.8	10.3	12.3	8.1	19.9	2.6	3.2	8.6	0.0	26.5	0.63	−0.06	0.99
Mi-We	9.6	5.7	9.0	11.1	6.1	6.8	2.3	10.8	17.6	0.0	21.1	0.99	−0.17	0.68
Mi-Ag	14.3	28.2	3.3	4.1	2.3	13.1	1.0	2.5	5.2	15.9	9.9	−0.13	0.99	−0.06
Mi-Wm	4.3	3.5	8.4	12.0	8.2	20.6	2.5	2.3	8.3	0.0	29.9	0.62	−0.03	0.99
Pb-We	7.8	8.0	9.4	12.0	5.7	6.4	2.4	9.8	17.5	0.0	21.1	0.99	−0.10	0.69
Pb-Ag	14.3	28.2	4.0	4.9	2.7	13.1	1.0	3.4	5.4	12.8	10.0	−0.06	0.99	−0.02
Pb-Wm	4.4	3.9	10.0	12.4	7.8	16.0	2.5	3.1	9.0	0.0	30.9	0.71	−0.06	0.99
Ro/Gy-We/Ag	9.3	7.0	9.3	12.0	6.2	6.5	2.4	9.9	16.9	0.0	20.4	0.99	−0.13	0.68
Ro/Gy-Ag	13.9	26.9	3.9	5.1	2.7	12.5	1.0	3.2	5.3	14.9	10.3	−0.09	1.00	−0.04
Ro/Gy-Wm/Ag	4.5	3.8	9.6	11.9	7.8	18.7	2.5	2.8	8.6	0.0	29.8	0.66	−0.04	1.00

**Table 4:** Amino acid composition of sample and reference materials expressed in w/w% of the selected amino acids.

AA (w/w%)	Ala	Gly	Val	Leu	Ile	Pro	Met	Ser	Phe	Asp	Hyp	Glu	*r* ^2^
Sample	9	6	9	12	6	5	3	12	4	14	0	19	
Egg	6	4	7	9	5	4	4	13	5	16	0	21	0.95
Animal glue	12	33	3	4	2	9	1	3	3	8	7	15	0.10
Milk	4	3	8	11	7	8	2	4	7	11	0	29	0.81

*Note.* Data from Colombini et al. [37]. Last column reports the correlation coefficient between amino acid composition of sample and proteinaceous binding media.
